# Prenatal risk factors for Tourette Syndrome: a systematic review

**DOI:** 10.1186/1471-2393-14-53

**Published:** 2014-01-30

**Authors:** Ting-Kuang Chao, Jing Hu, Tamara Pringsheim

**Affiliations:** 1 Department of Medicine, University of Alberta, Alberta, Canada; 2 Department of Clinical Neurosciences, University of Calgary, Calgary, Canada; 3 Alberta Children’s Hospital, C4-431, 2888 Shaganappi Trail NW, Calgary, AB T3B 6A8, Canada

**Keywords:** Tourette syndrome, Tic disorders, Prenatal morbidity, Risk factors

## Abstract

**Background:**

Tourette Syndrome (TS) appears to be an inherited disorder, although genetic abnormalities have been identified in less than 1% of patients, and the mode of inheritance is uncertain. Many studies have investigated environmental factors that might contribute to the onset and severity of tics and associated comorbidities such as obsessive compulsive disorder (OCD) and attention deficit hyperactive disorder (ADHD). A systematic review and qualitative analysis were performed to provide a broad view of the association between pre- and perinatal factors and TS.

**Methods:**

The Medline, Embase and PsycINFO databases were searched using terms specific to Tourette’s syndrome and keywords such as “pregnancy”, “prenatal”, “perinatal”, “birth” and “neonatal”. Studies were limited to studies on human subjects published in English or French through October 2012.

**Results:**

22 studies were included. Studies were of limited methodological quality, with most samples derived from specialty clinics, and most exposures ascertained retrospectively. The majority of the results for demographic factors of parents, including age, education, socioeconomic status, and marital status, revealed no significant association with the onset of TS, or the presence of comorbidity. Many factors were reported to be significantly associated with the onset of TS, the presence of comorbidity and symptom severity, but the most consistently reported factors were maternal smoking and low birth weight.

**Conclusions:**

There are few studies evaluating the relationship between pre and perinatal events and TS, and existing studies have major limitations, including the use of clinic rather than epidemiologically derived samples, retrospective data collection on pre and perinatal events and multiple hypothesis testing without appropriate statistical correction. The mechanism by which prenatal and perinatal adversities could lead to TS onset or symptom severity is unknown, but may be related to changes in the dopaminergic system as a result of early brain injury.

## Background

Tourette Syndrome (TS) appears to be an inherited disorder, although genetic abnormalities have been identified in less than 1% of patients, and the exact mode of inheritance has not been determined [1]. Some genetic studies have reported infrequent associations of specific genes with TS, including the mutation of SLITRK1 encoding SLIT and NTRK-like protein 1 and a rare functional mutation in the HDC gene encoding L-histidine decarboxylase [2-9]. While current research suggests that genetic factors confer the greatest risk for the development of TS, many studies have investigated environmental factors that might contribute to the onset of TS, associated comorbidities and more severe symptoms. Among these environmental factors, many studies report that pre- and perinatal factors may play an important role in the pathogenesis of TS [10-28]. However, analysis of these studies has revealed inconsistent results.

We performed a systematic review to gain a complete perspective on the association between pre- and peri-natal adversities and TS, including the onset of symptoms, symptom severity, and the presence and severity of comorbidities. There are no up to date systematic reviews focusing on this issue. In this article, a systematic review and qualitative analysis were performed to provide a broad view of the association between pre- and perinatal factors and TS.

## Methods

### Electronic searches and selection of studies

The Medline, Embase and PsycINFO databases were searched using terms specific to Tourette’s syndrome and keywords such as “pregnancy”, “prenatal”, “perinatal”, “birth” and “neonatal” (Additional file 1). Studies were limited to studies on human subjects published in English or French through October 2012. Two reviewers independently screened the titles and abstracts. Review articles, commentary articles, editorials, animal studies, and case-reports were excluded. Potentially relevant articles for full-text review were identified by reading abstracts and titles. Full text review was performed on any study for which the abstract or title suggested that the study evaluated pre or perinatal risk factors associated with the development of TS or related to TS severity. Disagreements were resolved by consensus between the two reviewers. Full texts of the selected articles were further reviewed and the following criteria were used to include articles for data extraction: 1) studies specific factors in the prenatal and perinatal period 2) evaluates association with the diagnosis or symptom severity of Tourette syndrome or its comorbidities 3) original study with fully published article available.

### Data extraction

The following information was extracted onto piloted forms: 1) First author, year and geographic location of the study 2) case definition and ascertainment, sources and numbers of cases and controls, matching factors 3) severity scales for Tourette’s syndrome or its comorbidities 4) specific risk factors 5) study results, including risk estimate and indicators of statistical significance.

## Results

**Figure 1:**
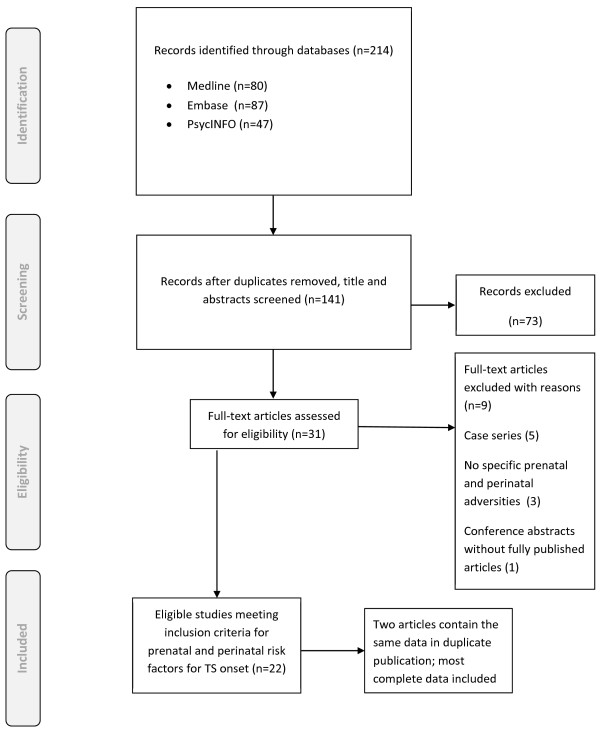
PRISMA flow diagram of included studies.

The electronic searches yielded 141 abstracts, with 31 abstracts selected for full-text review [10-40]. After full text review of these 31 articles, 9 were excluded because only case series were studied [31,32,35,37,38], only poster abstract was available [33], or the contents didn’t include pre- or peri-natal adversities [10,34,40]. Because of the extreme diversities among studies, the included 22 articles were further categorized according to the different purpose, study design, investigated factors, and outcome. In these 22 articles, two of them were duplicates of data [11,14]. Therefore, the data in one report was selected according to the completeness of data contained in both reports [11] (see Figure 1).

Of the 21 studies reporting original data, three were prospective cohort studies, nine were case control studies, four were single group cross sectional studies evaluating disease severity, one was a cross-sectional study with matched case–control pairs, three were twin or family studies, and one was a multiple case group comparative study. The three prospective cohort studies collected pre- and perinatal data prospectively. Two case control studies used pre- and perinatal data recorded on registered birth certificates at the time of delivery, and one cross-sectional study examined children for minor physical anomalies. The remaining fifteen studies collected pre- and perinatal risk factor data retrospectively through parental interview. With the exception of the three prospective cohort studies and one case control study, all samples of cases were derived from speciality clinic settings. See Table 1 for information on study design, sources of cases and controls, data collection and risk factors studied.

**Table 1:** Characteristics of included studies

**Study**	**Study design**	**Cases**	**Controls**	**Source of cases**	**Sources of controls**	**Matching factors**	**Diagnosis of TS**	**Data source**	**Prospective or retrospective collection of data on perinatal events**	**Risk factors evaluated**
Atladottir 2007 [29]	Cohort study	TS	Non-TS	Danish Medical Birth Registry cohort	Danish Medical Birth Registry cohort		ICD-10 in Psychiatric Registry	Registry	Prospective	Season of birth
Atladottir 2007 [29]	Cohort study	TS	Non-TS	Danish Medical Birth Registry cohort	Danish Medical Birth Registry cohort		ICD-10 in Psychiatric Registry	Registry	Prospective	Birth year (1990–1991, 1992–1993, 1994–1995)
Bos-Veneman 2010 [27]	Single group cross-sectional; comparison of tic and ADHD severity in those with/without risk factors	TS (62), Chronic motor (12) or vocal (1) tics	-	Psychiatry clinic (60) or TS Association (15)	-		DSM-IV-TR	Parent interview and questionnaire	Retrospective	Pregnancy complications, Delivery complications, First-week postnatal complications, Prenatal smoking exposure, Prenatal alcohol exposure
Bos-Veneman 2010 [27]	Single group cross-sectional; comparison of symptom severity in those with/without risk factors and genetic factors	Tic disorders	-	Psychiatry clinic (86) or TS Association (24)	-		DSM-IV-TR	Parent interview and questionnaire	Retrospective	(1) Genetic factors (Dopamine receptor D4 (DRD4) and interaction with environmental factors; (2) Pregnancy complications: HT, DM, infections, (pre)eclampsia, psychosocial stress; (3) Delivery complications: meconium-stained amniotic fluid, premature rupture of the membranes, nuchal cord, fetal bradycardia, placenta previa, artificial delivery; (4) Smoking during pregnancy
Burd 1999 [11]	Matched Case control (1:5)	TS	Healthy	North Dakota TS registry	North Dakota Health Department Registry	Sex, year of birth, month of birth	DSM-III, DSM-III-R, DSM-IV	Birth certificate data	Prospective	Birth weight, Apgar scores, mother's and father’s age and education, month prenatal care began, number of prenatal visits, gestational age
Csabi 2008 [12]	Cross-sectional study with matched case control pairs (1:1)	TS	Healthy	Outpatient clinic	Local elementary schools	Sex, age, ethnical origin	DSM-IV	Mehes Scale for evaluation of minor physical anomalies	Prospective	Presence of minor physical anomalies on examination
Hyde 1992 [25]	Twin study; evaluated differences between twins in presence of TS and tic severity	TS	-	Tourette Syndrome Association	-	Twins	Diagnostic interview	Mother completed questionnaire; confirmation of data through review of medical records where possible (not stated how many this was possible for)	Retrospective	Birth weight (n = 13 pairs), birth order (n = 8 pairs), handedness (n = 7 pairs), medical events and environmental factors (n = 13 pairs)
Kano 2002 [36]	Family study; probands and first degree relatives	TS		Not stated	First degree relatives		DSM-III	Report from mother	Retrospective	Premature delivery, use of forceps, apnea at birth, cyanosis at birth, slowed heartbeat
Khalifa 2005 [13]	Matched Case control (1:1)	TS	Healthy	Total population of children in a town in central Sweden	Total population of children in a town in central Sweden	Sex, age, school	DSM-IV	Interview with parents and medical record review	Retrospective	reduced optimality in the pre-, peri-, and neonatal periods (modified Prechtl's non-optimal score)
Klug 2003 [14] (duplicate data as Burd 1999) [11]	Matched Case control (1:5)	TS	Healthy	North Dakota TS registry	North Dakota Health Department Registry	Sex, year of birth, month of birth	Not stated	Birth certificate data	Prospective	Birth weight, Apgar scores, mother's and father’s age and education, month prenatal care began, number of prenatal visits, gestational age, number of child malformations
Kondo 1992 [15]	Case control	TS	Duchenne dystrophy	Neurology Outpatient Clinic	Other hospitals	None stated	Not stated	Patient and mother interview	Retrospective	Prenatal, perinatal and developmental history
Leckman 1990 [24]	Single group cross-sectional; comparison of tics severity in those with/without risk factors	TS	-	TS Specialty Clinic	-		DSM-III	Interview with mother; confirmation with obstetric records in 61%	Retrospective	41-item Obstetric Complications Scale (OCS), Perinatal complication scale (PCS), Level of Stress severity, Coping Ability Scale, 41-item Life Stress `Scale (LSS)
Leckman 1987 [16]	Twin study	Monozygotic twins disconcordant for TS	Unaffected twin	Not stated		Twins	Not stated	Parental interview	Retrospective	Birth weight
Mathews 2006 [26]	Single group cross-sectional; comparison of tics severity in those with/without risk factors	TS	-	TS specialty clinics; TS Association; Health care professionals, media advertisements, schools	-		DSM-IV	Parental interview	Retrospective	Prenatal problems, perinatal problems, and in utero medication exposure (details in the article)
Motlagh 2010 [17]	Case control	TS, TS + ADHD	Healthy	TS specialty clinic and TS Association	Telemarketing lists	Age, ZIP codes	DSM-IV	Maternal interview	Retrospective	Prenatal, obstetric, medical risk factors, life circumstances during pregnancy, birth weight, perinatal adverse events
Pasamanick 1956 [18]	Matched Case control (1:1)	Children with tics	Healthy	Child Psychiatry clinic	Birth register of the Bureau of Vital Records of the Baltimore City Health Department	Same place of birth, race, sex, maternal age group	Not stated	Hospital medical record of birth	Prospective	Pregnancy history, length of labour, complications of pregnancy and delivery, birth weight, condition of child during neonatal period
Saccomani 2005 [23]	Case control	TS, chronic tics	Healthy	Outpatient clinic	Offspring of hospital personnel	None stated	DSM-IV-TR	Parental interview	Retrospective	Definition of pre- and perinatal events: threatened miscarriage, prematurity, prolonged labour, umbilical cord around the neck, forceps delivery, and neonatal jaundice
Santangelo 1994 [19]	Multiple case group comparison; comparison of presence of comorbid OCD in those with/without risk factors	TS, TS + OCD		TS Association			DSM-III-R	Maternal interview	Retrospective	(1) pregnancy complications, (2) delivery complications, (3) Medications/procedures, and (4) Coffee, cigarettes, alcohol
Shimada 2012 [20]	Case Control	TS	General population data	Psychiatry outpatient clinic	General population database		DSM-IV-TR	Parent interview and questionnaire	Retrospective	Parental age, education level of parents, use of assistive reproduction, birth weight, gestational age
Pringsheim 2009 [21]	Nested case–control	TS + ADHD	TS	TS Specialty clinic	TS Specialty clinic		DSM-IV-TR	Parental interview and questionnaire	Retrospective	Maternal smoking, Maternal alcohol exposure, Low BW, Premature, small for GA, Breathing problems at birth, Maternal HT, Operative delivery, prenatal drug use,
Iida 1996 [22]	Case control	TS + OCS	TS	Outpatient psychiatry clinic	Outpatient psychiatry clinic		DSM-III-R	Maternal interview	Retrospective	Perinatal complications (postnatal jaundice, cord around neck, breech birth, premature birth)
Whitaker 1997 [39]	Prospective cohort study	Low birth weight children		Regional birth cohort of low birth weight children			DSM-III-R	Psychiatric diagnosis assessed at age 6 by structured diagnostic interview	Prospective	This was a prospective cohort study of low birth weight babies who were examined at age 6 for psychiatric disorders including TS. The study examined the relationship between neonatal cranial ultrasound abnormalities in these children and the diagnosis of psychiatric disorders at age 6.

### Studies investigating the association between pre- and perinatal factors and the onset of Tourette syndrome, and/or risk of comorbidity

There were 14 articles investigating individual pre- or peri-natal factors associated with the onset of TS, risk for comorbidities such as ADHD or OCD, or different genders in TS [11-23]. The summarized results are shown in Table 2. Because of diverse methods for measurement of the risk factors among studies, the results are presented according to the original definition by each study. Similar factors were grouped together but quantitative summarization was not possible because of small sample sizes and the diversity of studies.

**Table 2:** Pre- and peri-natal factors associated with the onset of Tourette’s syndrome (TS), and the presence of comorbidity

**Factors**	Results (Cases vs. Controls)*	**P value**	**Case number**	**Control number**	**Note**	**Reference**
***Demographic factors***						
Mother's age	25.69 (5.29) vs. 25.12 (5.2)	p = 0.33	92	460	Matched analysis	Burd 1999 [11]
	20 (1.4) vs. 23 (3.4)	**p < 0.01**	25	25		Khalifa 2005 [13]
	29.8 (4.5) vs. 29.6		66	1174999	Adjusted for birth year	Shimada 2012 [20]
	TS: 28.9 (4.4), TS + ADHD: 28.1 (5.3), Control: 28.3 (4.1)	NS	TS: 45, TS + ADHD:60	65		Motlagh 2010 [17]
Father's age	28.87 (6.25) vs. 27.78 (5.96)	p = 0.11	92	460	Matched analysis	Burd 1999 [11]
	31.9 (5.0) vs. 32.7	NS	65	1174999	Adjusted for birth year	Shimada 2012 [20]
	TS: 30.9 (5.0), TS + ADHD: 30.2 (6.2), Control: 30.4 (5.0)	NS	TS: 45, TS + ADHD:60	65		Motlagh 2010 [17]
Mother's education	12.90 (2.11) vs. 12.64 (1.92)	p = 0.24	92	460	Matched analysis	Burd 1999 [11]
Father's education	12.69 (2.44) vs. 12.67 (2.15)	p = 0.96	92	460	Matched analysis	Burd 1999 [11]
Parent's education	TS: 15.5 (2.0), TS + ADHD: 14.9 (1.8), Control: 15.3 (2.0)	NS	TS: 45, TS + ADHD:60	65		Motlagh 2010 [17]
Mean socioeconomic status	TS: 50.4 (2.7), TS + ADHD: 47.4 (10.6), Control: 48.5 (10.7)	NS	TS: 45, TS + ADHD:60	65		Motlagh 2010 [17]
Socio-economic status, parental education or divorce rate	No difference (no data provided)	NS	25	25		Khalifa 2005 [13]
Marital status	4/92 vs. 36/460	p = 0.93	92	460		Klug 2003 [14]
***Pre-conception condition***						
Major diseases before pregnancy or pre-existing medical conditions	(20/42 vs. 21/43) OR = 0.95, 95% CI = 0.41-2.23	p = 0.91	42	43		Kondo 1982 [15]
	TS vs. Control (1/45 vs. 4/65); TS + ADHD vs. Control (4/60 vs. 4/65)	NS	TS: 45, TS + ADHD:60	65		Motlagh 2010 [17]
***Pre-natal period***						
Month prenatal care began	2.47 (1.27) vs. 3.01 (1.40)	**p = 0.001**	92	460	Matched analysis	Burd 1999 [11]
Prenatal visits	10.00 (3.57) vs. 8.51 (3.22)	**p < 0.001**	92	460	Matched analysis	Burd 1999 [11]
Prenatal care begun after first trimester	OR = 0.49 (0.27-0.90), (14/92 vs. 123/460)	**p = 0.03**	92	460		Burd 1999 [11]
Minor physical anomalies (numbers)	5.46 (2.15) vs. 1.11(1.18); Mann–Whitney U-test 49.50, -Z = -4.92	**p = 0.001**	24	24		Csabi 2008 [12]
Number of prenatal problems	Presence of ADHD, OR = 2.97, 95% CI: 1.27-6.94	**p = 0.02**	180			Mathews 2006 [26]
Disordered pregnancies	(12/42 vs. 15/43) OR = 0.75, 95% CI = 0.30-1.87	p = 0.53	42	43		Kondo 1982 [15]
Any pregnancy complication	Male TS vs. Female TS: 63% vs. 83%	NS	46 M	7 F		Santangelo 1994 [19]
	TS + OCD vs. TS: 67% vs. 65%	NS	15	34		Santangelo 1994 [19]
One or more pregnancy complication	17/51 vs. 9/51 (OR = 2.33, 95% CI = 0.92-5.89)	p = 0.07	51	51		Pasamanick 1956 [18]
	TS + ADHD: 27/60, Control: 16/65	**p = 0.01**	TS + ADHD:60	65		Motlagh 2010 [17]
	TS: 17/45, Control: 16/65	NS	TS: 45	65		Motlagh 2010 [17]
Maternal hypertension	TS + ADHD vs. TS, OR = 1.04 (95% CI: 0.44-2.42)	p = 0.93	181	172	Unadjusted OR	Pringsheim 2009 [21]
Severe psychosocial stress	TS vs. Control (10/45 vs. 5/62, OR = 2.6, 95% CI = 0.08-8.7, p = 0.11); TS + ADHD vs. Control (11/55 vs. 5/62, OR = 3.1, 95% CI = 0.9-11.1, p = 0.07)	p = 0.11 and 0.07	TS: 45, TS + ADHD:60	65	Adjusted for gender	Motlagh 2010 [17]
Antibiotics exposure in pregnancy	TS: 11/45, TS + ADHD: 11/60, Control: 6/65	both **p < 0.05**	TS: 45, TS + ADHD:60	65		Motlagh 2010 [17]
Medication/procedures	Male TS vs. Female TS: 37% vs. 50%	NS	46 M	7 F		Santangelo 1994 [19]
	TS + OCD vs. TS: 27% vs. 44%	NS	15	34		Santangelo 1994 [19]
Number of medications exposed to in utero	Presence of ADHD, OR = 0.50, 95% CI: 0.25-1.00	**p < 0.05**	180			Mathews 2006 [26]
Maternal smoking (>10 cigarettes/d)	TS vs. Control (3/45 vs. 1/62, OR = 4.6, 95% CI = 0.45-46.6, p = 0.19); TS + ADHD vs. Control (7/60 vs. 1/62, OR = 8.5, 95% CI = 0.97-75.2, p = 0.052)	p = 0.19 and **0.05**	TS: 45, TS + ADHD:60	65	Adjusted for gender	Motlagh 2010 [17]
Maternal smoking	TS + ADHD vs. TS, OR = 2.43 (95% CI: 1.23-4.82)	**p = 0.01**	181	172	Adjusted OR	Pringsheim 2009 [21]
Maternal smoking	Presence of OCD (OR = 8.27, 95% CI = 0.87-78.20)	p = 0.07	180			Mathews 2006 [26]
Maternal alcohol exposure	TS + ADHD vs. TS, OR = 0.81 (95% CI: 0.46-1.41)	p = 0.45	181	172	Adjusted OR	Pringsheim 2009 [21]
Coffee, cigarettes, alcohol	Male TS vs. Female TS: 47% vs. 50%	NS	46 M	7 F		Santangelo 1994 [19]
	TS + OCD vs. TS: OR = 5.0, 95% CI: 1.3-19.4	**p < 0.05**	15	34		Santangelo 1994 [19]
***Perinatal period***						
Disordered deliveries	(16/42 vs. 14/43) (OR = 1.27, 95% CI = 0.52-3.11)	p = 0.59	42	43		Kondo 1982 [15]
Any delivery complication	Male TS vs. Female TS: OR = 9.4, 95% CI: 1.01-87.3	**p < 0.05**	46 M	7 F		Santangelo 1994 [19]
	TS + OCD vs. TS: 75% vs. 54%	NS	15	34		Santangelo 1994 [19]
Operative delivery	TS + ADHD vs. TS, OR = 1.10 (95% CI: 0.71-1.71)	p = 0.66	181	172	Unadjusted OR	Pringsheim 2009 [21]
Forceps delivery	Male TS vs. Female TS: 44% vs. 14%	NS	46 M	7 F		Santangelo 1994 [19]
	TS + OCD vs. TS: OR = 7.9, 95% CI: 3.2-19.5	**p < 0.01**	15	34		Santangelo 1994 [19]
Perinatal disorders: low BW, Asphyxia, severe vomiting, cyanosis, respiratory distress, jaundice, convulsion, high fever, incubator using, exchange transfusion	8/43 vs. 9/43, X^2 = 0.07 (OR = 0.86, 95% CI = 0.30-2.50)	NS (p = 0.79)	43	43		Kondo 1982 [15]
Perinatal complications (postnatal jaundice, cord around neck, breech birth, premature birth)	TS + OCS vs. TS only: 5/13 vs. 0/10)	**p < 0.01**	13	10	Fisher's exact test	Iida 1996 [22]
> 1 hypoxic event	TS vs. Control (17/45 vs. 18/65, OR = 1.4, 95% CI = 0.64-3.3, NS); TS + ADHD vs. Control (18/60 vs. 18/65, OR = 0.9, 95% CI = 0.4-2.2, NS)	NS	TS: 45, TS + ADHD:60	65	Adjusted for gender	Motlagh 2010 [17]
Apgar 1	7.170+/-1.909 vs. 7.517+/-1.623	p = 0.17	53	265	Matched analysis	Burd 1999 [11]
Apgar 5	8.396+/-1.446 vs.8.789+/-1.123	**p = 0.03**	53	265	Matched analysis	Burd 1999 [11]
Breathing problems at birth	TS + ADHD vs. TS, OR = 1.95 (95% CI: 0.87-4.37)	p = 0.10	181	172	Adjusted OR	Pringsheim 2009 [21]
***Combined pre- and peri-natal periods***						
Total with abnormalities of prenatal and perinatal periods	19/51 vs. 13/51 (OR = 1.74, 95% CI = 0.74-4.05)	p = 0.20	51	51		Pasamanick 1956 [18]
Reduced optimality in pre-, peri-, and neonatal periods	No statistical differences between cases and controls (no data provided) in reduced optimality		25	25		Khalifa 2005 [13]
Pre- and perinatal events	TS (26/48 (54%)), Chronic tics (24/48 (50%)) vs. Control (2/30 (6%)); TS: OR = 16.55 (95% CI = 3.54-77.40); Chronic tics: OR = 14.00 (95% CI = 3.00-65.44)	**p < 0.001** (both)	TS: 48, chronic tics: 48	30		Saccomani 2005 [23]
***Neonatal factors***						
Gestation age	39.64 (2.73) vs. 40.04 (3.54)	0.44	92	460	Matched analysis	Burd 1999 [11]
Gestational age (<37 week)	5/106 vs. 62289/1153363		106	1153363		Shimada 2012 [20]
	TS + ADHD vs. TS, OR = 3.83 (95% CI: 15.1-9.69)	**p < 0.01**	181	172	Unadjusted OR	Pringsheim 2009 [21]
Gestational age (<38 week)	TS: 1/45, TS + ADHD: 6/60, Control: 7/65	NS	TS: 45, TS + ADHD:60	65		Motlagh 2010 [17]
Gestational age (>42 week)	TS: 4/45, TS + ADHD: 4/60, Control: 2/65	NS	TS: 45, TS + ADHD:60	65		Motlagh 2010 [17]
Birth Weight	3342.2+/- 661.38 vs. 3447.7+/-607.71	p = 0.14	92	460	Matched analysis	Burd 1999 [11]
	TS: 3538+/-530, TS + ADHD: 3438+/-538, Control: 3519+/-755	NS	TS: 45, TS + ADHD:60	65		Motlagh 2010 [17]
	TS vs. Disconcordant Twin: 2228 + -244 vs. 2545 + -216	**p = 0.006**	6	6	Twins study (paired t test)	Leckman 1987 [16]
Low birth weight (<2500 g)	TS vs. Control (1/45 vs. 2/65, OR = 1.3, 95% CI = 0.11-15.4, NS); TS + ADHD vs. Control (3/57 vs. 2/65, OR = 0.55, 95% CI = 0.08-3.7, NS)	NS	TS: 45, TS + ADHD:60	65	adjusted for gender	Motlagh 2010 [17]
	TS vs. Control (1/116 vs. 9162/100097, OR = 0.094)		116	100097		Shimada 2012 [20]
	TS + ADHD vs. TS, OR = 2.74 (95% CI: 1.03-7.29)	**p = 0.04**	181	172	Adjusted OR	Pringsheim 2009 [21]
Small for gestational age (<10 percentile)	TS + ADHD vs. TS, OR = 1.13 (95% CI: 0.63-2.04)	p = 0.69	181	172	Unadjusted OR	Pringsheim 2009 [21]
Birth weight above 2500 g	Presence of ADHD, OR = 0.69, 95% CI: 0.52-0.92	**p = 0.02**	180			Mathews 2006 [26]
Birth order	X^2 = 2.53	NS	43	43		Kondo 1982 [15]
	First (78/116 vs. 53947/100118), second (34/116 vs. 34594/100118), > = third (8/116 vs. 11577/100118)		116	100118		Shimada 2012 [20]

*TS versus controls if not indicated. ADHD: Attention deficit hyperactive disorder; BW: birth weight; CI: confidence interval; NS: non-significant; OCD: Obsessive compulsive disorder; OCS: Obsessive compulsive symptoms; OR: odds ratio; TS: Tourette’s syndrome, statistically significant results shown in bold.

#### ***Demographic factors and pre-conception health status***

In Table 2, the majority of the results for demographic factors of parents, including age, education, socioeconomic status, and marital status, revealed no significant association with the onset of TS, or the presence of TS comorbidity such as ADHD [11,13,14,17,20]. There was only one small study (25 cases and 25 controls) in which the mother’s age in the group with TS was significantly younger than the control group (20 ± 1.4 versus 23 ± 3.4, p < 0.01) [13]. For pre-conception health status, major health problems prior to pregnancy were not associated with the onset of TS, or TS comorbidity, according to the results of two published studies [15,17].

#### ***Prenatal period***

During the prenatal period, many studies found that some prenatal adversities were significantly associated with the onset of TS or comorbidities, although the results were not consistent (Table 2). Burd (1999) reported that TS was associated with earlier prenatal care and more prenatal visits compared to the control group [11]. One cross sectional study evaluating the association between minor physical anomalies and TS suggested that early insults during pregnancy might be associated with the onset of TS, as children with TS had a significantly higher number of minor physical anomalies [12]. In 4 studies investigating the association between pregnancy complications and TS onset, the presence of comorbidity, or gender, one study showed a borderline association of one or more pregnancy complications with TS onset (OR = 2.33, 95% CI = 0.92-5.89, p = 0.069) [18]. Another study demonstrated that one or more pregnancy complications were significantly associated with TS with comorbid ADHD compared to controls, but not associated with TS only patients [17]. One study found that the overall number of prenatal problems was associated with a higher risk of ADHD comorbidity (OR = 2.97, 95% CI: 1.27-6.94, p = 0.02) [26].

Some individual and combined factors during pregnancy were also investigated, including maternal hypertension, psychosocial stress, antibiotic exposure, or medications/procedures [17,19,21]. In these studies, psychosocial stress was not significantly associated with the onset of TS or TS with ADHD compared to controls. In another study, antibiotic exposure was significantly associated with both TS and TS with ADHD (p = 0.02) [17].

With regard to maternal smoking, one study revealed borderline results in the association with onset of TS or TS with ADHD compared to the control group. The odds ratios were very high: TS (OR = 4.6), TS with ADHD (OR = 8.5) (Table 2) [17]. The study found that TS with ADHD had an even higher association with maternal smoking than TS only. This trend was also noted in another study- that compared to TS only patients, the presence of ADHD comorbidity was significantly associated with maternal smoking during the prenatal period (adjusted OR = 2.43, 95% CI; 1.23-4.82, p = 0.01) [21]. Maternal smoking was also reported to be associated with a borderline higher risk for OCD (OR = 8.27, 95% CI = 0.87-78.20, p = 0.07) [26]. In another study combining coffee, cigarettes, and alcohol exposures as one single factor, the presence of comorbid OCD with TS was significantly associated with these exposures compared to TS only patients (OR = 5.0, 95% CI: 1.3-19.4, p < 0.05) [19].

In summary, although there were no consistent results among studies, many factors were reported to be significantly associated with the onset of TS or the presence of comorbidities in TS patients. These factors included earlier prenatal care, more prenatal visits, pregnancy complications, antibiotic exposure, maternal smoking, and coffee, cigarette, and alcohol exposure. Prenatal adversities during a critical developmental stage may be associated with the onset of TS or the development of comorbidities in these patients.

#### ***Perinatal period***

The association between TS and perinatal risk factors was reported in 6 articles (Table 2) [11,15,17,19,21,22]. These studies suggest that while some perinatal risk factors were associated with specific conditions, the results are inconsistent. Among them, delivery complications were noted more frequently in boys with TS compared to girls (OR = 9.4, 95% CI: 1.01-87.3, p < 0.05) [19]. Forceps delivery was associated with the presence of OCD in TS patients compared to TS only (OR = 7.9, 95% CI: 3.2-19.5, p < 0.01) [19]. In addition, Iida reported that perinatal complications were associated with the presence of obsessive-compulsive symptoms in TS patients compared to TS only [22]. For the onset of TS, Burd et al. (1999) reported that the Apgar score at 5 minutes was significantly lower in children with TS compared to controls (8.396 ± 1.446 vs. 8.789 ± 1.123, p = 0.028) [11]. Although significant associations between TS and the perinatal factors described above were reported in these 6 articles, many of these articles revealed non-significant results for different measurements. Compared to prenatal factors, a relatively smaller proportion of results for perinatal factors revealed significant associations with TS.

#### ***Combined prenatal and perinatal period***

When prenatal and perinatal adversities were considered as one factor, only one of 3 studies revealed significant associations of prenatal and perinatal adversity with TS (OR = 16.55, 95%CI = 3.54-77.40, p < 0.001) or chronic tics (OR = 14.00 95%CI = 3.00-65.44, p < 0.001) (Table 2) [23]. However, in the other two studies, no associations were reported [13,18].

#### ***Neonatal factors***

The relationship between gestational age, birth weight, birth order and TS has been investigated in several studies (Table 2) [11,15-17,20,21]. Most studies reported non-significant results for these factors. In 4 articles investigating gestational age, only one study revealed that the presence of ADHD in TS patients was significantly associated with prematurity (<37 weeks) compared to TS only patients in an unadjusted evaluation (OR = 3.83, 95% CI: 15.1-9.69, p < 0.01) [21]. The same study also found that low birth weight (<2500 g) was associated with a higher odds of comorbid TS and ADHD (OR = 2.74, 95% CI: 1.03-7.29, p = 0.04) [21]. Another study found that higher birth weight was associated with a lower risk of ADHD comorbidity in TS patients (OR = 0.69, 95% CI: 0.52-0.92, p = 0.02) [26]. In a twin study where only one twin was affected by TS, lower birth weight was noted in the TS twin compared to their disconcordant twin without TS (TS twins: 2228 ± 244 gm vs. Twins without TS: 2545 ± 216 gm, paired t test, p = 0.006) [16]. For birth order, both studies found that it is not associated with the onset of TS [15,20].

### Studies investigating the association between pre- and perinatal factors and severity of tic symptoms and comorbidities

There were 5 articles investigating the association between pre- and perinatal factors and the severity of tic symptoms and comorbidities [24-28]. All articles included only TS patients using a single group cross sectional design with comparison of disease symptom severity between those exposed and not exposed to perinatal adversity. All of the significant and non-significant results in these articles were summarized in Table 3.

**Table 3:** Pre and perinatal factors associated with tic severity and severity of co-morbid ADHD, OCD, and other disorders

**Factors**	**Results (exposed vs. Non-exposed)**	**P value**	**Directions of association**	**Case Number**	**Scales of measurements**	**Reference**
***Pre-natal period***						
Pregnancy complications	Current YGTSS 22.3+/-9.4 vs. 17.3+/-8.4	**p = 0.03**	↑	70	YGTSS	Bos-Veneman 2010 [27]
	2.0 + -3.7 vs. 3.9 + -4.9	**p = 0.05**	↓	110	CYBOCS	Bos-Veneman 2010 [27]
	No difference in worst ever tic severity	p = 0.68	**-**	70	YGTSS	Bos-Veneman 2010 [27]
	No difference in ADHD severity	p = 0.45	**-**	65	ADHD rating scale	Bos-Veneman 2010 [27]
	No difference in tic severity	NS	-	110	YGTSS	Bos-Veneman 2010 [27]
	No difference in social behaviours	NS	-	110	CSBQ	Bos-Veneman 2010 [27]
	No difference in anxiety and depression	NS	-	110	RCADS	Bos-Veneman 2010 [27]
Severity of nausea/vomiting	Associated with current tic severity	**p = 0.01**	↑	31	TS Global scale and C-GAS (adjusted for gender)	Leckman 1990 [24]
Number of medications exposed in utero	Associated with increased global tic severity	**p < 0.00001**	↑	180	YGTSS	Mathews 2006 [26]
	Interference of OCS	p = 0.08	↑	180		Mathews 2006 [26]
Maternal smoking	ADHD Severity 26.4 + -10.1 vs. 19.1 + -10.9	**p = 0.05**	↑	70	ADHD rating scale	Bos-Veneman 2010 [27]
	Associated with more severe ADHD symptoms modified by positive family history of mental disorders	**p = 0.04**	↑	70	ADHD rating scale	Bos-Veneman 2010 [27]
	9.1 + -4.1 vs. 6.1 + -4.1	**p = 0.05**	↑	110	MDD RCADS	Bos-Veneman 2010 [27]
	6.6 + -3.8 vs. 4.4 + -3.7	**p = 0.05**	↑	110	CSBQ subscale orientation ratings	Bos-Veneman 2010 [27]
	Positive association with tic severity	**p < 0.00001**	↑	180	YGTSS	Mathews 2006 [26]
	Interference of OCS	**p = 0.03**	↑	180		Mathews 2006 [26]
	No difference in current tic severity	p = 0.38	**-**	70	YGTSS	Bos-Veneman 2010 [27]
	No difference in worst ever tic severity	p = 0.86	**-**	65	YGTSS	Bos-Veneman 2010 [27]
	No difference in tic severity	NS	-	110	YGTSS	Bos-Veneman 2010 [27]
	No difference in obsessive compulsive severity	NS	-	110	CYBOCS	Bos-Veneman 2010 [27]
	No difference in social behaviours other than orientation ratings	NS	-	110	CSBQ	Bos-Veneman 2010 [27]
	No difference in anxiety and total scale of RCADS	NS	-	110	RCADS	Bos-Veneman 2010 [27]
Maternal alcohol drinking	No difference in current tic severity	p = 0.13	**-**	70	YGTSS	Bos-Veneman 2010 [27]
	No difference in worst ever tic severity	p = 0.10	**-**	70	YGTSS	Bos-Veneman 2010 [27]
	No difference in ADHD severity	p = 0.71	**-**	65	ADHD rating scale	Bos-Veneman 2010 [27]
Psychological stress	Associated with current tic severity	**p = 0.03**	↑	31	TS Global scale and C-GAS	Leckman 1990 [24]
***Perinatal period***						
Delivery complications	Worst ever YGTSS 28.7+/-9.1 vs. 23.4+/-7.3	**p = 0.01**	↑	70	YGTSS	Bos-Veneman 2010 [27]
	Associated with lesser total YGTSS ratings after adjusting for age and gender modified by DRD4 3R allele	**p = 0.02**	↓	110	YGTSS	Bos-Veneman 2010 [27]
	Associated with higher total anxiety ratings and total RCADS after adjusting for age and gender modified by DRD4 2R allele	**p = 0.004 and 0.006**	↑	110	RCADS	Bos-Veneman 2010 [27]
	No difference in current tic severity	p = 0.82	-	70	YGTSS	Bos-Veneman 2010 [27]
	No difference in ADHD severity	P = 0.13	-	65	ADHD rating scale	Bos-Veneman 2010 [27]
	No difference in tic severity	NS	-	110	YGTSS	Bos-Veneman 2010 [27]
	No difference in obsessive compulsive severity	NS	-	110	CYBOCS	Bos-Veneman 2010 [27]
	No difference in social behaviours	NS	-	110	CSBQ	Bos-Veneman 2010 [27]
	No difference in anxiety and depression	NS	-	110	RCADS	Bos-Veneman 2010 [27]
Delivery complications and other perinatal adversities	No difference in tic severity between TS twins	NS	-	13 pairs	Shapiro scales	Hyde 1992 [25]
Severity of perinatal complications	Borderline associated with the tic severity	p = 0.07	**↑**	31	TS Global scale and C-GAS (adjusted for gender)	Leckman 1990 [24]
Number of perinatal complications	No association with the tic severity	NS	-	31	TS Global scale and C-GAS (adjusted for gender)	Leckman 1990 [24]
***Neonatal factors***						
Birth Weight	Higher tic scores in lighter body weight twin on both Shapiro and YGTSS scales	**p = 0.001 and 0.01**	↓	13 pairs	Shapiro scales and YGTSS	Hyde 1992 [25]
	Within-pair body weight difference is correlated with within-pair tic score difference (rho = 0.72)	**p < 0.005**	↓	13 pairs	Shapiro scale	Hyde 1992 [25]
Birth order in TS twins	Mean first-born tic score vs. mean second-born tic score: 7.1 + -6.5 vs. 8.4 + -5.6	p = 0.57	-	8 pairs	Shapiro scales and YGTSS	Hyde 1992 [25]
Handedness in TS twins	Tic score in right-handed vs. left-handed: 10.6 + -4.7 vs. 6.1 + -4.6	p = 0.13	-	7 pairs	Shapiro scales and YGTSS	Hyde 1992 [25]
***Postnatal period***						
First-week postnatal complications	No difference in current tic severity	p = 0.87	-	69	YGTSS	Bos-Veneman 2010 [27]
	No difference in worst ever tic severity	p = 0.74	-	69	YGTSS	Bos-Veneman 2010 [27]
	No difference in ADHD severity	p = 0.83	-	64	ADHD rating scale	Bos-Veneman 2010 [27]

ADHD: Attention Deficit Hyperactive disorder; C-GAS: Children's Global Severity Scale; CI: confidence interval; CSBQ: Children's Social Behavior Questionnaire; CYBOCS: Children's Yale-Brown Obsessive-Compulsive scale; DRD4: Dopamine receptor D4; MDD; Major Depression Disorder; NS: non-significant; OCD: Obsessive Compulsive Disorder; OCS: Obsessive-Compulsive Symptoms; OR: odds ratio; RCADS: Revised Child Anxiety and Depression Scale; TS Tourette’s syndrome; YGTSS: Yale Global Tic Severity Scale; Borderline (0.05 < = p <0.10) or significant (p < 0.05) results are marked with the association directions, statistically significant results shown in bold.

#### ***Prenatal factors***

Some prenatal factors were reported to be significantly associated with the severity of tic symptoms or comorbidities (Table 3). Pregnancy complications which included hypertension, infections, (pre)eclampsia, psychosocial stress, or diabetes mellitus were reported to be associated with greater tic severity at a single time point as measured by the Yale Global Tic Severity Scale (YGTSS) [27]. However, these pregnancy complications were found to be associated with lower mean compulsive CYBOCS ratings in these patients [28]. In addition, severity of nausea/vomiting during pregnancy was also noted to be associated with current tic severity as measured using the TS Global Scale based on all available information (direct examination, review of videotapes, parental- and self-reports, school reports, and medical records) [24]. With respect to in utero medication exposure, this was associated with global tic severity and a borderline higher risk for obsessive compulsive symptoms but a lower risk for symptoms of ADHD in individuals with TS [26].

In addition to the prenatal factors mentioned above, some studies have investigated the association of tic severity or comorbidity severity with maternal smoking, maternal alcohol use, psychological stress during pregnancy, and the overall number of prenatal problems (Table 3). More severe ADHD rating scale scores, measured with the parent version of the ADHD Rating Scale, were associated with maternal smoking (smoking versus non-smoking: 26.4 ± 10.1 versus 19.1 ± 10.9, p = 0.05) [27]. This association was more prominent when there was a positive family history of mental disorders (p = 0.04) [27]. In another study, higher depression and autism scale scores, measured with the major depressive disorder subscale of the revised Child Anxiety and Depression Scale (RCADS) and the parent version of the Children’s Social Behaviour Questionnaire (CSBQ), were noted in TS patients with a history of exposure to maternal smoking [28]. Maternal smoking was also reported to be associated with greater worst ever lifetime tic severity as measured by the YGTSS, and more obsessive compulsive symptoms in another study [26]. These results suggest an association between maternal smoking and tic, ADHD and OCD severity in individuals with TS. In addition to maternal smoking, maternal psychological stress was noted to be associated with greater tic severity [24]. With regard to the prenatal alcohol exposure, there was no association between maternal alcohol use and tic severity or ADHD comorbidity [27].

#### ***Perinatal factors***

The presence of delivery complications including meconium-stained amniotic fluid, premature rupture of the membranes, nuchal cord, fetal bradycardia, placenta praevia, or artificial delivery was reported to be associated with higher worst ever tic severity (presence versus absence: 28.7 ± 9.1 vs. 23.4 ± 7.3, p = 0.01) as measured by the YGTSS [27].

Dopaminergic genes are candidate genes in TS studies given that alterations in dopaminergic neurotransmission are believed to contribute to the etiology of tics. In a study investigating the interaction between the dopamine receptor D4 (DRD4) gene and perinatal factors associated with tic severity, TS patients with the DRD4 3R allele and a history of delivery complications had a lower total tic scale rating, measured with the YGTSS, compared to individuals without the DRD4 3R allele (p = 0.02) [28]. The same study also found that in patients with the DRD4 2R allele, higher total anxiety ratings and total Revised Child Anxiety and Depression Scale scores (RCADS) were noted in TS patients with delivery complications (p = 0.004 and 0.006 respectively). These results suggest an interaction of genetic and environmental factors on the severity of tics and associated comorbidities in TS patients.

#### ***Neonatal factors***

In a twin study evaluating birth weight and lifetime tic severity, the twin with lower birth weight had higher tic scores measured with Shapiro Symptom Check List and YGTSS [25]. In the same study, the within-pair body weight difference was linearly correlated with the within-pair tic score difference measured with the Shapiro score (rho = 0.72, p < 0.005) [25]. These findings suggested that lower birth weights were associated with the greater tic severity between identical twins. In addition, these associations had a linear dose–response relationship. These results suggest that lower birth weight is associated with higher tic scores in TS patients.

### Time trends and seasonal variation

There was one article investigating time trends in the incidence of Tourette syndrome in a population-based cohort [30]. In this study, a statistically significant increase was found in the cumulative incidence across specific birth years for TS from 1990 to 1995. For example, for children age 9, the cumulative incidences are 2.3 (95% CI: 1.4-3.1), 2.4 (95% CI: 1.5-3.2), and 3.9 (95% CI: 2.9-4.9) for birth cohort in 1990–1991, 1992–1993, and 1994–1995, respectively. The study could not identify if there were environmental factors including pre- or perinatal adversities associated with this increase in incidence, or whether this was due to an improvement in identification and diagnosis of tic disorders over this time period. Another study was performed using the same cohort data to evaluate the seasonal variation in the incidence of TS. The results found that there was no evidence of seasonal variation of birth and TS incidence [29].

### Other types of studies

Some indirect evidence also suggests that perinatal difficulties may play a role in the onset of TS. In one study investigating TS probands and relatives’ risks for TS, perinatal difficulties in TS probands were inversely related to the recurrence risk among first-degree relatives of TS probands [36]. This suggests that TS probands with perinatal problems had a lower risk of TS in first degree relatives. It provides indirect evidence that part of the risk for TS in these probands was related to these perinatal insults. In another study investigating the association between neonatal cranial ultrasound abnormalities and the prevalence of tic disorders in low-birth-weight children, the prevalence of tic disorders at age of 6 was significantly associated with parenchymal lesions and/or ventricular enlargement in neonatal cranial ultrasound examinations (OR = 8.7, 95% CI: 1.3-57.7, p = 0.02) [39]. These results suggest that neonatal brain injury might increase the risk for the onset of tic disorders.

## Discussion

Overall, there were few articles investigating the association between pre- and perinatal adversities and the onset and severity of TS, and the presence and severity of comorbid disorders. Although some studies have reported associations between pre- and perinatal factors and TS, many of the results are inconsistent. Taken as a whole, there is only very weak evidence to support that pre- and perinatal adversity are associated with the development of TS, TS symptom severity and the presence or severity of comorbidities.

One of the major issues regarding the available literature on the relationship between TS and pre and perinatal risk factors is the overall poor methodological quality of the studies performed to date. Only five studies evaluated data on perinatal events that was collected prospectively on registered birth certificates or as part of a birth registry. The majority of studies used clinically ascertained samples of individuals with TS, which may differ substantially in disease severity than those ascertained through population based means. The available data are therefore susceptible to bias.

In comparing the results of the case control studies using birth certificate data with those who ascertained information about perinatal events from mothers retrospectively, there is no clear difference in the strength of association between TS and various perinatal risk factors. Burd [11] reported significantly earlier prenatal care, more prenatal visits, a lower odds of having care begun after the first trimester (three highly related factors), and lower Apgar scores at 5 minutes among cases than controls. Pasamanick [18] did not detect any significant relationships between abnormalities during the pre or perinatal period and TS.

Of the five studies examining the relationship between perinatal adversity and TS and comorbidity symptom severity, the majority of positive results reported would not remain significant after appropriate statistical correction for multiple hypothesis testing. The exception is the finding reported by Matthews [26] of the association between increased global tic severity and the number of medications reported in utero and maternal smoking (both p < 0.00001).

Of all the risk factors studied, maternal smoking and low birth weight appear to be the only risk factor with consistent significant associations. Maternal smoking was reported to be associated with the presence of comorbid ADHD and OCD, and TS, ADHD and OCD symptom severity. Although these results suggest a connection between maternal smoking and the onset of TS, other factors linking these exposures to TS need to be considered. Nicotine has a therapeutic effect for some TS and ADHD patients [41-46]. Nicotine has been shown to reduce complex tics and improve behaviours related to inattention in TS patients [41]. Individuals with ADHD have a higher risk of smoking and nicotine dependence from adolescence to young adulthood [47]. If mothers with tics or ADHD smoke, the association of maternal smoking and TS in the offspring might be due to heritability of these disorders rather than smoking itself. Many individuals are unaware of their tics and are never diagnosed with TS and therefore may deny a family history of the disorder. This confounding effect should be carefully examined in future studies to clarify the association of prenatal smoking exposure and the onset or severity of TS, and the presence and severity of comorbid ADHD and OCD.

Most studies did not report significant associated neonatal factors with TS, except for low birth weight. Low birth weight was found to be associated with comorbid ADHD and tic severity. Low birth weight may be an independent factor associated with TS or comorbidities, or may be a consequence of prenatal adversity. Although this effect would be minimized in twin studies, further investigations are necessary to identify whether low birth weight itself is associated with TS or if it is the proxy of other prenatal insults.

The limitations of this systematic review include the overall poor methodological quality of the included studies, with differing study designs and analytic methods, and extreme diversity of measurements among different studies. Definitions of prenatal or perinatal adversities differed between studies, and were most commonly retrospectively ascertained. Finally, as the clinical symptoms of TS wax and wane during the disease course, the measurement of tic severity at a single point of time does not truly represent disease severity for patients. Although it is difficult to draw a valid conclusion from this literature, many pre- and perinatal adversities were reported to be associated with the onset of TS, the presence of comorbidities, and the severity of tics and comorbidities. Future studies are likely to make a significant contribution, especially if they include population derived cohorts, use data on perinatal events that was obtained prospectively, and use methods to control for multiple hypothesis testing. Standards for defining pre and perinatal events should be adhered to, and TS symptom severity should be measured at multiple time points if this outcome is being considered.

The mechanism through which prenatal and perinatal events lead to TS onset or worsening tic severity is unknown. These factors could contribute to microscopic abnormalities in brain structure or function that lead to the development of tics. TS is a neurodevelopmental disorder which shares common features with autism and attention deficit hyperactivity disorder. Dysfunction of the dopaminergic system has been implicated in all three disorders, and evidence from animal studies suggest that prenatal stress may cause changes in the dopaminergic system [48-51]. The frequent comorbidity of these neurodevelopmental disorders in clinical practice suggests a common pathophysiological basis, with shared prenatal and perinatal risk factors in autism and ADHD supporting these relationships [52-54].

## Conclusions

In conclusion, this systematic review provides weak evidence for an association between pre- and perinatal adversity and the onset of TS, comorbid disorders, and the severity of tics, ADHD and OCD symptoms. Of all the risk factors studied, maternal smoking and low birth weight appear to be most consistently implicated. Studies of higher methodological quality are needed in order to better understand the relationship between prenatal and perinatal events and TS.

## Competing interests

Ting-Kuang Chao, Jing Hu and Tamara Pringsheim declare that they have no competing interests.

## Authors’ contributions

TKC performed the data extraction and synthesis, wrote the initial draft of the manuscript, and made subsequent revisions to the manuscript. JH created and executed the search strategy for original research articles, reviewed all abstracts for inclusion criteria, and assisted with revision of the manuscript. TP obtained grant funding for the study, created the search strategy for original research articles, reviewed all abstracts for inclusion criteria, and revised the manuscript. All authors read and approved the final manuscript.

## Pre-publication history

The pre-publication history for this paper can be accessed here:

http://www.biomedcentral.com/1471-2393/14/53/prepub

## Supplementary Material

Additional file 1 Medline search strategy. Click here for file

## References

[B1] Bloch M State M Pittenger C Recent advances in Tourette syndrome Curr Opin Neurol 2011 24 2 119 25 10.1097/WCO.0b013e328344648c 21386676 PMC4065550

[B2] Abelson JF Sequence variants in SLITRK1 are associated with Tourette's syndrome Science 2005 310 5746 317 20 10.1126/science.1116502 16224024

[B3] Ercan-Sencicek AG L-histidine decarboxylase and Tourette's syndrome N Engl J Med 2010 362 20 1901 8 10.1056/NEJMoa0907006 20445167 PMC2894694

[B4] Verkerk AJ CNTNAP2 is disrupted in a family with Gilles de la Tourette syndrome and obsessive compulsive disorder Genomics 2003 82 1 1 9 10.1016/S0888-7543(03)00097-1 12809671

[B5] Chou IC Association of the Slit and Trk-like 1 gene in Taiwanese patients with Tourette syndrome Pediatr Neurol 2007 37 6 404 6 10.1016/j.pediatrneurol.2007.06.017 18021920

[B6] Deng H Examination of the SLITRK1 gene in Caucasian patients with Tourette syndrome Acta Neurol Scand 2006 114 6 400 2 10.1111/j.1600-0404.2006.00706.x 17083340

[B7] Fabbrini G A large Italian family with Gilles de la Tourette syndrome: clinical study and analysis of the SLITRK1 gene Mov Disord 2007 22 15 2229 34 10.1002/mds.21697 17712845

[B8] Orth M Autosomal dominant myoclonus-dystonia and Tourette syndrome in a family without linkage to the SGCE gene Mov Disord 2007 22 14 2090 6 10.1002/mds.21674 17702041

[B9] Zimprich A Sequence analysis of the complete SLITRK1 gene in Austrian patients with Tourette's disorder Psychiatr Genet 2008 18 6 308 9 10.1097/YPG.0b013e3283060f6f 19018236

[B10] Alexander GM Peterson BS Testing the prenatal hormone hypothesis of tic-related disorders: gender identity and gender role behavior Dev Psychopathol 2004 16 2 407 20 15487603 10.1017/s095457940404458x

[B11] Burd L Prenatal and perinatal risk factors for Tourette disorder J Perinat Med 1999 27 4 295 302 10560082 10.1515/JPM.1999.042

[B12] Csabi G Minor physical anomalies in Tourette syndrome Eur J Psychiatry 2008 22 3 173 180

[B13] Khalifa N Von Knorring AL Tourette syndrome and other tic disorders in a total population of children: Clinical assessment and background Acta Paediatr, Int J Paediatr 2005 94 11 1608 1614 10.1111/j.1651-2227.2005.tb01837.x 16352498

[B14] Klug MG A comparison of the effects of parental risk markers on pre- and perinatal variables in multiple patient cohorts with fetal alcohol syndrome, autism, Tourette syndrome, and sudden infant death syndrome: an enviromic analysis Neurotoxicol Teratol 2003 25 6 707 17 10.1016/j.ntt.2003.07.018 14624970

[B15] Kondo K Nomura Y Tourette syndrome in Japan: etiologic considerations based on associated factors and familial clustering Adv Neurol 1982 35 271 6 6959498

[B16] Leckman JF Nongenetic factors in Gilles de la Tourette's syndrome Arch Gen Psychiatry 1987 44 1 100 10.1001/archpsyc.1987.01800130112025 3467660

[B17] Motlagh MG Severe psychosocial stress and heavy cigarette smoking during pregnancy: an examination of the pre- and perinatal risk factors associated with ADHD and Tourette syndrome Eur Child Adolesc Psychiatry 2010 19 10 755 764 10.1007/s00787-010-0115-7 20532931 PMC3932440

[B18] Pasamanick B Kawi A A study of the association of prenatal and paranatal factors with the development of tics in children; a preliminary investigation J Pediatr 1956 48 5 596 601 10.1016/S0022-3476(56)80095-4 13307362

[B19] Santangelo SL Tourette's syndrome: what are the influences of gender and comorbid obsessive-compulsive disorder? J of the Am Academy of Child & Adolesc Psychiatry 1994 33 6 795 804 10.1097/00004583-199407000-00004 8083136

[B20] Shimada T Parental age and assisted reproductive technology in autism spectrum disorders, attention deficit hyperactivity disorder, and Tourette syndrome in a Japanese population Res Autism Spectrum Disord 2012 6 1 500 507 10.1016/j.rasd.2011.07.010

[B21] Pringsheim T Prenatal and perinatal morbidity in children with Tourette syndrome and attention-deficit hyperactivity disorder J Dev Behav Pediatr 2009 30 2 115 21 10.1097/DBP.0b013e31819e6a33 19322105

[B22] Iida J The clinical features of Tourette's disorder with obsessive-compulsive symptoms Psychiatry Clin Neurosci 1996 50 4 185 9 10.1111/j.1440-1819.1996.tb02740.x 9201774

[B23] Saccomani L Tourette syndrome and chronic tics in a sample of children and adolescents Brain Dev 2005 27 5 349 52 10.1016/j.braindev.2004.09.007 16023550

[B24] Leckman JF Perinatal factors in the expression of Tourette's syndrome: An exploratory study J Am Acad Child Adolesc Psychiatry 1990 29 2 220 226 10.1097/00004583-199003000-00010 1969861

[B25] Hyde TM Relationship of birth weight to the phenotypic expression of Gilles de la Tourette's syndrome in monozygotic twins Neurology 1992 42 3 Pt 1 652 8 1549232 10.1212/wnl.42.3.652

[B26] Mathews CA Association between maternal smoking and increased symptom severity in Tourette's syndrome Am J Psychiatr 2006 163 6 1066 73 10.1176/appi.ajp.163.6.1066 16741208

[B27] Bos-Veneman NG Role of perinatal adversities on tic severity and symptoms of attention deficit/hyperactivity disorder in children and adolescents with a tic disorder J Dev Behav Pediatr 2010 31 2 100 106 10.1097/DBP.0b013e3181cc7cbc 20110829

[B28] Bos-Veneman NGP Minderaa RB Hoekstra PJ The DRD4 gene and severity of tics and comorbid symptoms: main effects and interactions with delivery complications Mov Disord 2010 25 10 1470 6 10.1002/mds.23122 20629147

[B29] Atladottir HO Variation in incidence of neurodevelopmental disorders with season of birth Epidemiology 2007 18 2 240 5 10.1097/01.ede.0000254064.92806.13 17202868

[B30] Atladottir HO Time trends in reported diagnoses of childhood neuropsychiatric disorders: a Danish cohort study Arch Pediatr Adolesc Med 2007 161 2 193 8 10.1001/archpedi.161.2.193 17283306

[B31] Eapen V Clinical features and associated psychopathology in a Tourette syndrome cohort Acta Neurol Scand 2004 109 4 255 60 10.1046/j.1600-0404.2003.00228.x 15016007

[B32] Fernandez-Mayoralas DM Fetal alcohol syndrome, Tourette syndrome, and hyperactivity in nine adopted children Pediatr Neurol 2010 43 2 110 6 10.1016/j.pediatrneurol.2010.03.008 20610121

[B33] Griffiths BJ Hafez A Stern JS Month of birth of people attending a tourette syndrome clinic J Neurol Neurosurg Psychiatry 2010 81 10 e14

[B34] Hyde TM Electroencephalographic abnormalities in monozygotic twins with Tourette's syndrome Br J Psychiatry 1994 164 6 811 817 10.1192/bjp.164.6.811 7952989

[B35] Incagnoli T Kane R Developmental perspective of the Gilles de la Tourette syndrome Percept Mot Skills 1983 57 3 Pt 2 1271 81 6582444 10.2466/pms.1983.57.3f.1271

[B36] Kano Y Leckman JF Pauls DL Clinical characteristics of Tourette syndrome probands and relatives' risk J Am Acad Child Adolesc Psychiatry 2002 41 10 1148 1149 10.1097/00004583-200210000-00005 12364837

[B37] Micheli F Gilles de la Tourette syndrome: Clinical features of 75 cases from Argentina Behav Neurol 1995 8 2 75 80 24487424 10.3233/BEN-1995-8202

[B38] Shapiro AK Shapiro E Wayne H Birth, developmental, and family histories and demographic information in Tourette's syndrome J Nerv Ment Dis 1972 155 5 335 44 4117160 10.1097/00005053-197211000-00005

[B39] Whitaker AH Psychiatric outcomes in low-birth-weight children at age 6 years: relation to neonatal cranial ultrasound abnormalities Arch Gen Psychiatry 1997 54 9 847 56 10.1001/archpsyc.1997.01830210091012 9294376

[B40] Zelnik N Height distribution in children with Tourette syndrome J Child Neurol 2002 17 3 200 4 10.1177/088307380201700309 12026236

[B41] Howson AL Clinical and attentional effects of acute nicotine treatment in Tourette's syndrome Eur Psychiatry 2004 19 2 102 12 10.1016/j.eurpsy.2003.11.002 15132126

[B42] Levin ED Nicotine effects on adults with attention-deficit/hyperactivity disorder Psychopharmacology (Berl) 1996 123 1 55 63 10.1007/BF02246281 8741955

[B43] McConville BJ The effects of nicotine plus haloperidol compared to nicotine only and placebo nicotine only in reducing tic severity and frequency in Tourette's disorder Biol Psychiatry 1992 31 8 832 40 10.1016/0006-3223(92)90315-Q 1643197

[B44] Potter AS Newhouse PA Acute nicotine improves cognitive deficits in young adults with attention-deficit/hyperactivity disorder Pharmacol Biochem Behav 2008 88 4 407 17 10.1016/j.pbb.2007.09.014 18022679

[B45] Silver AA Case study: long-term potentiation of neuroleptics with transdermal nicotine in Tourette's syndrome J Am Acad Child Adolesc Psychiatry 1996 35 12 1631 6 10.1097/00004583-199612000-00015 8973070

[B46] Silver AA Transdermal nicotine and haloperidol in Tourette's disorder: a double-blind placebo-controlled study J Clin Psychiatry 2001 62 9 707 14 10.4088/JCP.v62n0908 11681767

[B47] Fuemmeler BF Kollins SH McClernon FJ Attention deficit hyperactivity disorder symptoms predict nicotine dependence and progression to regular smoking from adolescence to young adulthood J Pediatr Psychol 2007 32 10 1203 13 10.1093/jpepsy/jsm051 17602186

[B48] Baier CJ Gestational restraint stress and the developing dopaminergic system: an overview Neurotox Res 2012 22 1 16 32 10.1007/s12640-011-9305-4 22215534

[B49] Makkonen I Effects of fluoxetine treatment on striatal dopamine transporter binding and cerebrospinal fluid insulin-like growth factor-1 in children with autism Neuropediatrics 2011 42 5 207 9 22015434 10.1055/s-0031-1291242

[B50] Rogers TD Reorganization of circuits underlying cerebellar modulation of prefrontal cortical dopamine in mouse models of autism spectrum disorder Cerebellum 2013 12 4 547 556 10.1007/s12311-013-0462-2 23436049 PMC3854915

[B51] Staal WG de Krom M de Jonge MV Brief report: the dopamine-3-receptor gene (DRD3) is associated with specific repetitive behavior in autism spectrum disorder (ASD) J Autism Dev Disord 2012 42 5 885 8 10.1007/s10803-011-1312-z 21691864 PMC3324694

[B52] Gardener H Spiegelman D Buka SL Perinatal and neonatal risk factors for autism: a comprehensive meta-analysis Pediatrics 2011 128 2 344 55 10.1542/peds.2010-1036 21746727 PMC3387855

[B53] Guinchat V Pre-, peri- and neonatal risk factors for autism Acta Obstet Gynecol Scand 2012 91 3 287 300 10.1111/j.1600-0412.2011.01325.x 22085436

[B54] Latimer K Disruptive behaviour disorders: a systematic review of environmental antenatal and early years risk factors Child Care Health Dev 2012 38 5 611 28 10.1111/j.1365-2214.2012.01366.x 22372737

