# Supplementary material for: Prenatal risk factors for Tourette Syndrome: a systematic review
Source: BMC Pregnancy Childbirth. 2014 Jan 30;14:53. doi: 10.1186/1471-2393-14-53 (PMC4015943; doi:10.1186/1471-2393-14-53)
Supplement: Additional file 1 — Medline search strategy. [file 1471-2393-14-53-S1.docx]

**Additional file 1: Medline search strategy**

1. pregnancy.mp.

2. prenatal.mp.

3. perinatal.mp.

4. birth.mp.

5. neonatal.mp.

6. 1 or 2 or 3 or 4 or 5

7. exp tic disorders/

8. exp tics/

9. Tourette.tw.

10. 7 or 8 or 9

11. 6 and 10

12. limit 11 to (english or french)

13. limit 12 to animals

14. 12 not 13
